# Artificial intelligence-assisted laser ultrasound method for the estimation of porosity in hairpin weld seams^☆^

**DOI:** 10.1016/j.pacs.2025.100770

**Published:** 2025-10-08

**Authors:** Markus Saurer, Guenther Paltauf, Oliver Spitzer, Tobias Reitmayr, Gordana Djuras, Birgit Kornberger, Ulrike Kleb, Robert Nuster

**Affiliations:** a Department of Physics, University of Graz, Universitaetsplatz 5, Graz, 8010, Austria; b Miba Automation Systems, Aurachkirchen 45, Aurachkirchen, 4812, Austria; c POLICIES – Institute for Economic, Social and Innovation Research, JOANNEUM RESEARCH, Leonhardstraße 59, Graz, 8010, Austria

**Keywords:** Laser ultrasound, Weld seam inspection, Hairpin stator, Artificial intelligence

## Abstract

Hairpin technology is being used as a replacement for the traditional winding stator in electric motors. In hairpin stator manufacturing, copper rods are used to achieve a higher slot fill factor. These rods are joined together in pairs through laser welding, forming a closed circuit. However, this welding process is prone to air inclusions in the welds, which can negatively impact the efficiency and durability of the motor. The present study aims to estimate the total volume of these air inclusions using laser ultrasonic measurements. Laser ultrasound is a fast, non-contact, non-destructive method that can cope with the limited sample accessibility, making it ideal for inline testing of these weld seams. To evaluate the effectiveness of laser ultrasound, a stator was intentionally manipulated prior to laser welding to favor the formation of air inclusions. The porosity of the weld seams was determined through computed tomography images. It was demonstrated that due to the complex geometry of the hairpin welds, leading to a complex ultrasound wave field, standard methods to estimate the porosity from laser ultrasound B-scans are difficult to apply. As an alternative approach, an algorithm that is based on artificial intelligence was utilized for the purpose of estimating the air inclusion volume in the welds from laser ultrasonic measurements. The outcomes demonstrated a median correlation of 0.6 between this estimate and the pore volume obtained from the computed tomography data, despite the utilization of only 48 samples. Moreover, these results were evaluated against a model where the labels were randomly mixed, and highly informative regions regarding pore volume were identified in the B-scans, which have the potential to accelerate the process of acquiring data.

## Highlights

• Laser ultrasound applicability for defect detection in hairpin welds demonstrated.
• Artificial intelligence methods utilized to estimate porosity in hairpin welds.
• Comparison of computed tomography with laser ultrasound results.
• Regions in B-scans highlighted that are most informative for neural networks.

## 1 Introduction

Nowadays, hairpin stators are replacing traditional winding stators in electric motors due to their higher slot fill factor, resulting in a lower DC resistance. This leads to a higher efficiency, especially at lower rotation frequencies. At high rotation frequencies, the higher AC losses in hairpin stators diminish the higher efficiency. Another advantage of hairpin technology is the superior management of heat and the enhanced reproducibility of the production process [1], [2]. Hairpin stators use copper rods (pins) instead of wires to conduct the electric current. These rods have the shape of a “U” and are assembled into the stator core according to the required winding scheme. To form a closed circuit, the pins are twisted and contacted by joining neighboring pins after the twisting process with laser-welding [3], [4]. Each weld seam must be intact to avoid the possibility of a defective stator. The important quality parameter of such a weld seam is the cross-sectional area between the two pins. This area should not be much smaller than the cross-sectional area of the individual pin to avoid current bottlenecks in the circuit. The possible causes for reducing this area can be divided into two categories. One concerns anomalies in the weld geometry, such as insufficient weld depth, and the other concerns internal defects in the form of air inclusions that can form during the laser welding process as described by Zhao et al. [5] and Pastor et al. [6]. Both types of defects must be avoided in order to ensure the quality of the weld and thus the quality, efficiency and durability of the stator. For faster and cost-effective weld defect detection, it is advantageous if the methods used can be implemented in-line and are non-contact.

Optical methods have been used to detect the geometric defects, as recently reported in the literature. Raffin et al. [7] and Vater et al. [8] used image-based neural networks to predict the weld quality, and Mayer et al. [9], [10] additionally estimated the cross-sectional area in hairpin welds. Will et al. [11] used optical coherence tomography to investigate the influence of the misalignment of the individual pins prior to laser welding on the welding result, and Yu et al. [12] discussed the adaptation of welding strategies to reduce the influence of those misalignments that could be optically detected prior to the welding process. Besides geometry defects, optical methods are also used to detect other types of welding defects such as spatters as reported by Hartung et al. [13], [14] and Heider et al. [15].

Other methods for estimating weld depth and weld porosity are based on the analysis of laser welding process parameters such as laser power, feed rate, welding paths, beam diameter, etc. as reported by Darwish et al. [16], D’Arcangelo et al. [17] and Dimatteo et al. [18]. As shown by Darwish et al. [16] and D’Arcangelo et al. [17], porosity is difficult to estimate from process parameters due to the different pore formation mechanism [19]. Two examples, where high speed computed tomography has been used to investigate this pore formation mechanism, are the work of Katayama et al. [20] on aluminum butt welds and the work of Omlor et al. [21] on hairpin welds.

Efforts to reduce the probability of the formation of air inclusions in welds have been described in the literature. Kamimuki et al. [22] showed that side gas can be used to reduce porosity in bead-on-plate welding of stainless steel sheets. Omlor et al. [23] and Zhu et al. [24] recently modified laser welding parameters like scan paths, laser power and scan speed to reduce the porosity in hairpin welds. In addition, Ning et al. [25] reported that a standing ultrasonic wave field can be used to stabilize weld formation in hairpins.

In contrast to estimating the pore volume from weld geometry or process parameters, mechanical strength [17], electrical resistance [11], micrographs, computed tomography, eddy currents and classical ultrasound have recently been used to directly detect air inclusions in welds [19]. However, all of these current methods for direct detection of air inclusions are unsuitable for non-contact in-line detection due to either their destructive nature, their long process time, or the limited sample accessibility. Therefore, this work proposes the use of laser ultrasound (LUS) for the in-line detection of air inclusions in hairpin welds. LUS is a fast, non-contact material testing technique that can be used from a large stand-off distance. In this process, the ultrasound waves are generated by pulsed laser radiation. The time-resolved detection of the waves at specific locations is conducted by analyzing a phase change or deflection of the reflected probe beam. The use of lasers for both the excitation and detection of ultrasonic waves is quite convenient, since the same laser or beam path can be used as for laser welding.

As demonstrated by Pelivanov et al. [26], the porosity of samples can be estimated through the analysis of the backscattered ultrasound energy from voids. In a previous study [27], these concepts were expanded to small samples with curved surfaces, where the ultrasonic field experiences a pronounced interaction with the sample boundaries. In this setting, the detection of air inclusions with LUS in samples mimicking weld seams has been investigated in a more defined environment (i.e., drilled holes in a milled sample with a curved surface extending indefinitely in one dimension). Additionally, Chandler et al. [28] recently showed that the analysis of classical ultrasound data enables the accurate identification of defective, geometrically complex samples by using the data from the pristine sample as a reference.

The fundamental distinction in the present application is that for hairpin welds no reference can be provided because each weld possesses a unique and complex geometry. This includes process-related variations in the gap between the two pins and in the surface curvature. This unique geometry gives rise to echoes and surface acoustic waves (SAWs) that are geometry-dependent and interfere with signals from the air inclusions inside of the weld. A relative reduction in the energy of the SAW can be achieved through the utilization of large excitation laser spots, a concept previously explored by Saurer et al. [29]. Recent studies by Davis et al. [30] have proposed other potential solutions to suppress SAWs for large, flat surface samples, which are difficult to apply to the small samples with highly complex geometries considered in this study. In contrast to Pelivanov et al. [26] and Saurer et al. [27], where more straightforward temporal separation of geometry-dependent echoes and SAWs from echoes of voids was possible, this study requires the identification of more sophisticated time windows for extracting porosity information from LUS measurements. The optimization problem for finding these time windows possesses a multitude of variables.

It is therefore convenient to employ alternative data analysis tools based on artificial intelligence (AI). AI tools have already been successfully utilized for the analysis of LUS signals. In their respective studies, Lv et al. [31] and Guo et al. [32] used AI techniques to estimate the dimensions of subsurface defects. Keshmiri et al. [33] demonstrated the efficacy of AI in reducing the number of measurement points required for defect detection in plate structures. Mei et al. [34] illustrated the potential of AI in addressing some limitations of the Full Matrix Capture Total Focusing Method for reconstructing complex-shaped defects. In this work, the usage of AI methods is demonstrated to overcome the limitations of LUS when applied to geometrically complex samples, where SAWs and multiple reflections from the unique sample boundaries interfere with the reflections from the defects.

The structure of the present work is as follows. In Section 2, the hairpin stator fabricated for this study is introduced, followed by the results of a computed tomography (CT) analysis, which provides a reference for quantifying air inclusions within the weld seams. Subsequently, the LUS measurement setup is described. Section 3 describes the problems of standard methods to estimate the porosity in hairpin weld seams from LUS measurements and the usability of AI methods. Finally, the results are summarized and discussed.

## 2 Materials and methods

### 2.1 Materials

**Fig. 1:**
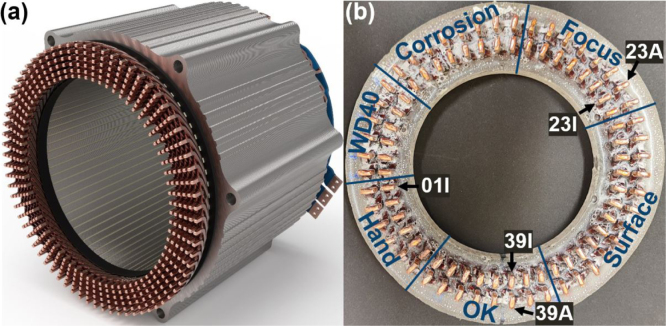
(**a**) Schematic picture of a hairpin stator. (**b**) Photo of cut-out section of the hairpin stator used for the LUS examinations.

To investigate the applicability of LUS, a copper hairpin stator (symbolic picture in Fig. 1(a)) was produced with some previous manipulations to enhance the formation of air inclusions during the welding process. To facilitate sample handling, only the section of the stator containing the weld seams was excised and embedded in epoxy, as illustrated in Fig. 1(b). The cross sectional area of an unwelded pin pair was approximately 6.3 mm × 2.5 mm. The stator comprised two rows of pin pairs, with each row consisting of 48 pin pairs, resulting in a total of 96 weld seams. Each weld seam was assigned a unique identifier comprising a number and the letter “I” for the inner ring and “A” for the outer ring. The aforementioned labeling is illustrated for five examples (01I, 23I, 23 A, 39I, 39 A) in Fig. 1(b). Furthermore, the regions where different manipulations were carried out prior to laser welding were labeled as follows. Region “Hand”: The pin pair was touched by hand prior to the laser welding process. Region “WD40”: Prior to laser welding, the pin pair was wetted with creeping oil (WD-40). Region Focus: The laser beam for laser welding was defocused. Region “Corrosion”: Prior to laser welding, the copper was exposed to corrosion. Region “Surface”: Before laser welding, the surface of the pin pair was roughened. Region “OK”: No manipulation was conducted in this region prior to laser welding.

### 2.2 Computed tomography inspection

**Fig. 2:**
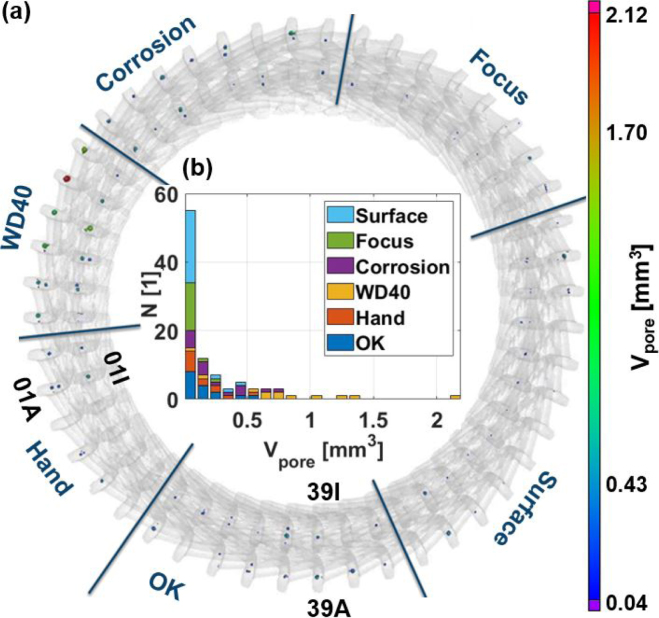
(**a**) CT data of the hairpin stator, with regions of differing manipulations prior to laser welding (labeled in Fig. 1(b)). The color of each inclusion denotes its respective size. (**b**) Statistic of total pore volume in the welds. The x-axis of the bar chart is divided into regions with a width of 0.1 mm^3^ with the first bar including the amount of weld seams with a total pore volume between [0 mm^3^–0.1 mm^3^). The colors used in this image represent the different manipulation regions.

Computed tomography (CT) has already been established as an investigative tool to examine porosity in laser-welded seams [19]. In Fig. 2(a) the CT data of the stator under consideration is shown. The voxel volume is 80 × 80 × 80 μm^3^. The colored data points correspond to the volume of the air inclusions, which have been extracted from the data using VGEasyPore software, ranging from 0.04 up to 2.12 mm^3^. In Fig. 2(b) the distribution of total pore volumes (sum over all air inclusions in one weld seam) within the hairpin welds is depicted. The different colors in this picture correspond to the different manipulation regions prior to the laser welding process (labeled in Fig. 1(b)).

It can be observed that weld seams containing large air inclusions with a volume of over 0.4–0.5 mm^3^ are underrepresented. This is approximately the upper limit of acceptable pore volumes for the given use case. Furthermore, the region “WD40” appears to be particularly susceptible to the presence of large air inclusions. However, there are also two welds in this region, namely 03I and 08I, where only small air inclusions formed during the laser welding process. It is also noteworthy that the welds in the region “Focus” are not prone to the presence of significant air inclusions. This may be attributed to the specific characteristics of the welding process employed in this region. Nevertheless, the weld quality in this region is not sufficient due to the reduced cross sectional area. Another interesting observation is that surface roughening enhances the quality of the welding results as the region “Surface” exhibits the lowest mean pore volume, apart from the region “Focus”. The total pore volumes ($V_{pore,real}$) derived from the CT analysis served as the ground truth for subsequent analysis.

### 2.3 Laser ultrasound inspection

The application of conventional reconstruction methods, such as the Synthetic Aperture Focusing Technique (SAFT), to LUS data is challenging when applied to samples with a curved surface and limited size. This is due to the presence of strong artifacts resulting from multiple reflections in the B-scans, as previously demonstrated [27]. Consequently, the subsequent LUS analysis will be limited to estimating the total volume of the air inclusions in these welds, as opposed to reconstructing their position. The estimate from LUS measurements will be correlated with the air inclusion volume extracted from the CT data.

**Fig. 3:**
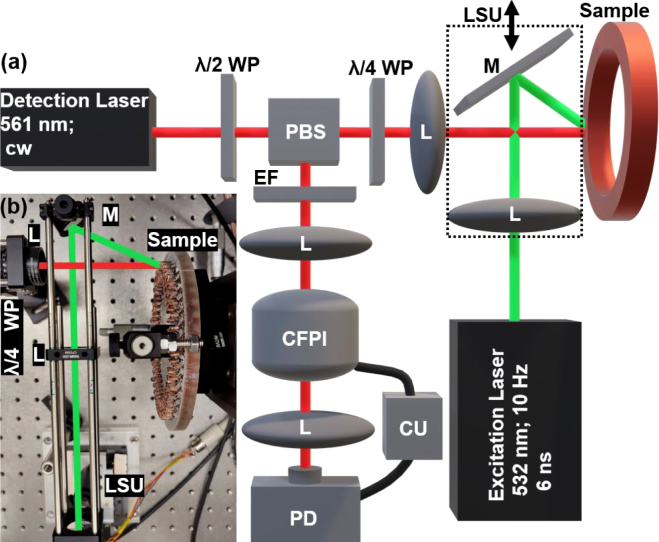
(**a**) Schematic sketch of the setup used for LUS investigations. (**b**) Picture of LUS setup and sample holder. PBS...Polarizing Beam Splitter, M...Mirror, CU...Control Unit, PD...Photo Diode, CFPI...Confocal Fabry–Perot Interferometer, LSU...Linear Shifting Unit, WP...Wave Plate, L...Lens, EF...Edge Filter.

The LUS experiments were conducted using a setup based on the methodology described by Murfin et al. [35] and Dewhurst et al. [36]. In the employed detection scheme, the frequency shift of the reflected light from the surface of the sample due to ultrasound waves was analyzed with a Confocal Fabry–Pérot Interferometer (CFPI), as illustrated in Fig. 3. The pulse energy used for excitation was 4 mJ, and the power of the detection laser reached up to 170 mW. A concise overview of the laser parameters is provided in Fig. 3(a) and more details can be found in previous works [27], [29]. In order to perform a B-scan, the mirror and the lens for guiding the excitation laser pulse onto the surface of the sample were mounted on a linear shifting unit (LSU), thereby enabling the distance between the excitation laser spot and the detection laser spot to be varied. Due to the slight ablative nature of LUS generation, which was needed to maximize the ultrasound energy in the sample, the excitation laser beam could not be placed at the same position as the detection laser spot on the sample surface, as in this case the detection scheme would have become unstable. Additionally, at this position, stray light would severely interfere with the measurement of the actual signal. Despite the slight ablative effect, it can be assumed that the wave propagation characteristics are dominated by thermoelastic generation, as evidenced by the imperceptible alterations observed on the sample surface post-measurement, where the surface appeared to be polished rather than scratched. In the context of hairpin welds, this minimal ablation does not pose a significant issue, as it does not result in the introduction of scratches or steps into the material. To prevent significant changes in beam diameter on the sample surface during scanning, a lens with a large focal length was utilized, and the sample surface was placed at a distance, where the excitation laser beam spot diameter was approximately 1 mm. This relatively large diameter was previously identified as preferable for detecting air inclusions in this type of sample [29]. The size of the detection laser spot on the surface of the sample was around 7 μm. The low-frequency component of the DC output, below 100 kHz, was used to actively stabilize the cavity of the interferometer. The measurement quantity, the AC output of the photodiode, is proportional to the velocity of the surface displacement of the sample caused by the ultrasound waves. In addition to applying a low pass filter of 20 MHz, the detection frequency range was influenced by the frequency transfer function of the CFPI, as was demonstrated in Dewhurst et al. [36]. The used CFPI with a Finesse > 200 has a peak response at around 5 MHz and a −3 dB bandwidth from 2.5 MHz to 8.2 MHz.

## 3 Results

**Fig. 4:**
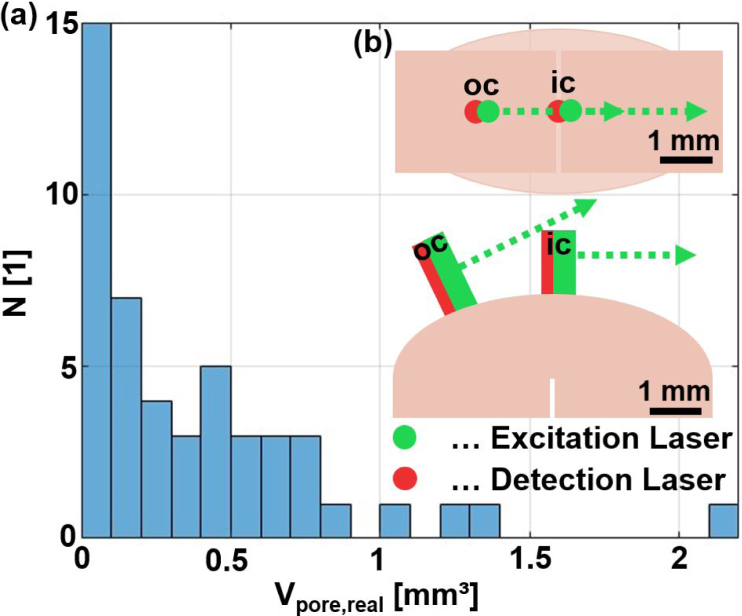
(**a**) Statistics of total volume of air inclusions $V_{pore,real}$ in the weld seams used in the LUS investigations. (**b**) Schematic sketch of the two LUS measurement configurations ic and oc.

The 16 weld seams from the region “Focus” (labeled in Fig. 1(b)) could be identified to have geometric defects and were therefore excluded from further analysis. Of the remaining 80 weld seams, 48 were subjected to LUS investigation. As the statistics in Fig. 2(b) demonstrate, the data set is relatively limited in size, and the large inclusion volumes are underrepresented. The 48 samples were chosen such that this imbalance is reduced, leading to the new statistics presented in Fig. 4(a). The underrepresentation of samples with large air inclusions remains, albeit to a lesser extent.

Each measurement consisted of two B-scans with two different detection positions and with 30 different excitation positions, moving the LSU from 0.1 to 3 mm with a step size of 0.1 mm, where 0 mm approximately equals the fixed position of the detection laser spot. One detection position was located in the center of the weld seam (ic), and the other detection position was located in the center of the inner pin (oc). A schematic of this measurement configuration is shown in Fig. 4(b). The purpose of changing the excitation laser spot position on the sample surface was to modify the ultrasound energy distribution within the sample, as outlined in [29]. The optical detection required the sensing laser beam to always be perpendicular to the surface of the weld seam. Therefore, to move to position oc in Fig. 4(b), the sample was rotated relative to the fixed laser beam. For the weld seam samples with less perfect surfaces, in some cases some rotation was also necessary for the detection position ic in order to obtain a better detection signal strength. As was shown in [27], the large excitation laser spot results in the amplitude of the surface displacement due to SAWs being comparable with the amplitude of the surface displacement due to echoes from the bulk, therefore reflections of SAWs at surface irregularities become negligible.

### 3.1 Data analysis with standard methods

**Fig. 5:**
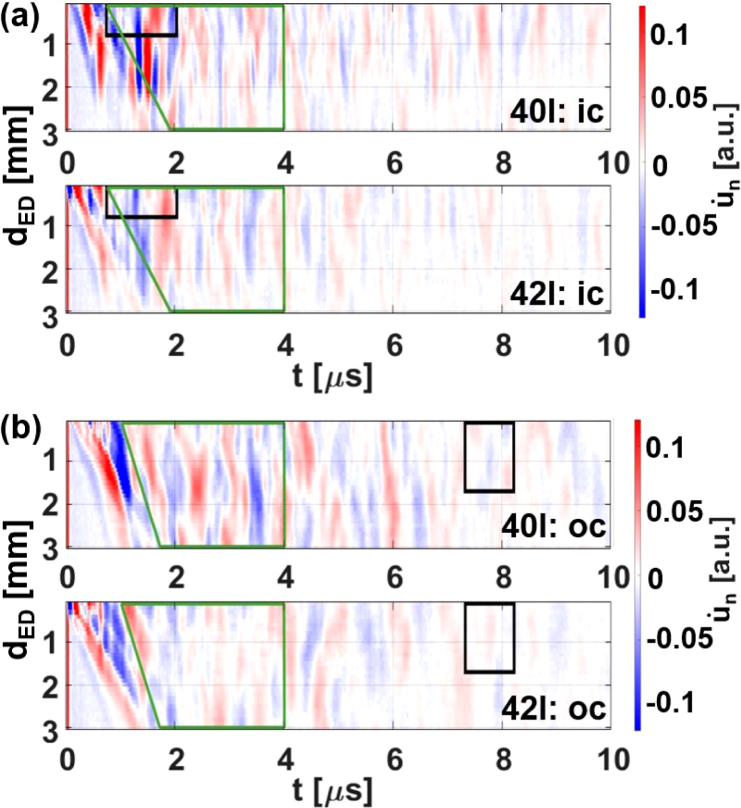
Comparison of B-scans from two weld seams from the region “OK” (labeled in Fig. 1(b)) with different inclusion volumes (40I : 0.6 mm^3^, 42I: 0.1 mm^3^). In (**a**) the measurement configuration ic from Fig. 4(b) was used and in (**b**) the measurement configuration oc. Additionally two different windows (green and black) used for further analysis in Fig. 7 are visible. t corresponds to the time, $d_{ED}$ to the distance between excitation and detection and $\overset{˙}{u_{n}}$ to the surface displacement in normal direction.

Fig. 5(a) shows B-scans from two weld seams with different air inclusion sizes (Pin 40I: 0.6 mm^3^, Pin 42I: 0.1 mm^3^) obtained by using the measurement configuration ic (see Fig. 4(b)). A comparison reveals that the variation in the B-scan from the weld seam with the larger inclusion (Pin 40I) is significantly higher. Additionally, there is a high-intensity structure in the B-scan of the sample 40I at around 1.5 μm strongly indicating the presence of the large air inclusion. In Fig. 5(b) the B-scans for the measurement configurations oc can be seen. There, the same trend as for the configuration ic can be observed. This phenomenon of the higher variation in the B-scans with higher pore volume was introduced by Pelivanov et al. [26] and was previously extended to small, curved surface samples in a related work using aluminum weld seam models [27]. In both of these studies, the outer geometry of the samples was identical on the length scale of the typical ultrasonic wavelength. Consequently, it was possible to introduce a reference and exclude the structures in the B-scans that lacked information about the internal quality of the sample. This approach led to correlation coefficients reaching 0.9 between a measure of the variation in the B-scans (E-value) and the volume of the air inclusions (drilled holes) [27].

**Fig. 6:**
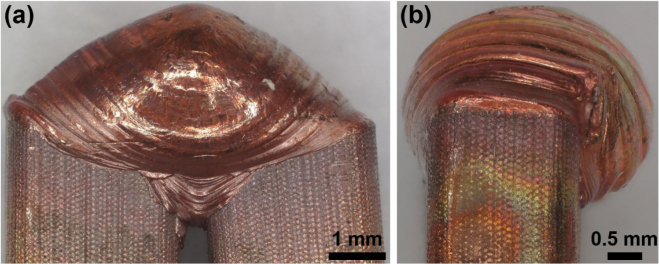
Two examples for geometric ultrasound scatterers in hairpin welds: (**a**) Gap between the two pins. (**b**) Weld seam hangs over the pins.

In the present study, it is more challenging to differentiate between structures observed in B-scans that originate from the sample geometry and those that originate from air inclusions. This is due to the fact that each weld seam has a unique geometry, leading to unique excitation conditions and to unique echoes stemming from this particular geometry. In addition to the unique surface, there are geometrical ultrasound scatterers, such as the gap between the two pins and the boundaries where the weld seam widens beyond the original pin geometry, resulting in overhangs. One extreme example of each of those two geometric ultrasound scatterers is depicted in Fig. 6. In image (a) the gap is shown and in image (b) the overhang. Video S1 in the supplementary material shows numerical simulations of the 3D wave propagation in such a sample, where both the ultrasound scattering at the air inclusions and at the overhang can be observed and distinguished by comparing simulations with air inclusions (first column) with simulations without air inclusions (second column).

Despite these additional ultrasound scatterers and the complex and unique geometry, an initial back-scattering energy analysis, similar to those conducted in Pelivanov et al. [26] and in a previous work [27] was performed. In accordance with the aforementioned works, a time window was selected in which the geometry-dependent structures in the B-scans, such as surface waves, were excluded. This was achieved by defining the time window for analysis to start after the arrival of the surface waves. Given the inherent variability in the surfaces of the samples, the average of all B-scans is employed to identify the average structures from the surface waves. The end point of the designated time window was chosen such that direct echoes from air inclusions in the entire volume of the weld seam (≈3 mm × 2.5 mm × 6.3 mm) can reach the detection laser, which was around 4 μs, assuming a transverse wave velocity of 2260 m/s for copper. The transverse wave velocity was utilized due to its status as the predominant mode for the excitation conditions employed, as previously observed in related studies [27], [29] and as discussed by Pyzik et al. [37]. The resulting time window is indicated in the B-scan images for both detection positions (ic and oc) with a green frame in Fig. 5.

**Fig. 7:**
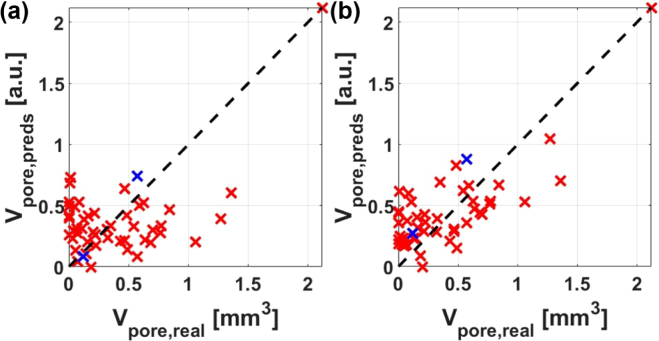
Correlation between air inclusion volume $V_{pore,real}$ and back-scattered energy $V_{pore,LUS}$. (**a**) When using the green window from Fig. 5 the correlation coefficient is 0.52 for all samples and 0.04 when excluding the sample with the largest air inclusion volume. (**b**) When using the black window from Fig. 5 the correlation coefficient is 0.61 and 0.78 respectively. The blue crosses correspond to the samples 40I and 42I with inclusion volumes of around 0.6 and 0.1 mm^3^ respectively. The B-scans of these samples can be seen in Fig. 5.

In Fig. 7(a), the measure of variation ($V_{pore,LUS}$: sum of absolute square) inside the green time window in the B-scans (Fig. 5) is plotted versus the real pore volumes extracted from the CT data, showing a correlation of 0.52 and 0.04, respectively, when neglecting the sample with the largest pore volume. The observed amplitude variability of the B-scan images shown in Fig. 5 is higher for sample 40I ($V_{pore,real}=0.6\;{\mathrm{mm}}^{3}$) compared to sample 42I ($V_{pore,real}=0.1\;{\mathrm{mm}}^{3}$). This observation can be further substantiated by comparing the two values of the blue crosses in 7(a). However, the selection of these two samples was deliberate, and the data in this figure indicate that, in general, the implementation of these standard methods poses significant challenges for samples that are both unique and possess complex shapes.

Lowpass frequency filtering of the signals with cutoffs around 10 MHz prior to the back-scattered energy analysis, as suggested in [26], did not show any improvements. This is because the frequency filter function employed has a similar shape to the transfer function of the transmission mode of the CFPI, as described in Dewhurst et al. [38]. Furthermore, any normalization of the A-scans prior to the signal energy analysis, such as to the SAW signal amplitude, proved to be counterproductive in terms of maximizing the correlation between porosity and LUS energy. This is primarily due to the interference of the SAW structures with echoes stemming from the interior of the sample, which are similar in amplitude due to the large excitation laser spot on the sample surface. However, optimization of the correlation coefficient can be achieved by not utilizing the simple time window (green window in Fig. 5) for the back-scattering energy analysis. Instead, as Fig. 7(b) demonstrates, time windows (black rectangles in Fig. 5) can be found where higher values of correlation between air inclusion volume and back scattered energy are achieved, reaching up to 0.78 and 0.61, depending on whether the sample with the largest pore volume is included or not. For this optimization, twelve parameters were introduced. Eight of these parameters were designated for the start and end position on the time- and $d_{ED}$ - axis for both measurement configurations (ic and oc). The remaining four parameters were designated for the two weighting factors and two exponents in the summation of the back scattered energy values of both measurement configurations. In general, more parameters could be introduced to enhance the correlation. However, this optimization process is laborious when performed manually and lacks proper generalizability. Furthermore, when standard gradient or non-gradient-based techniques are employed, the outcomes are significantly influenced by the initial guess.

### 3.2 Data analysis with artificial intelligence methods

**Fig. 8:**
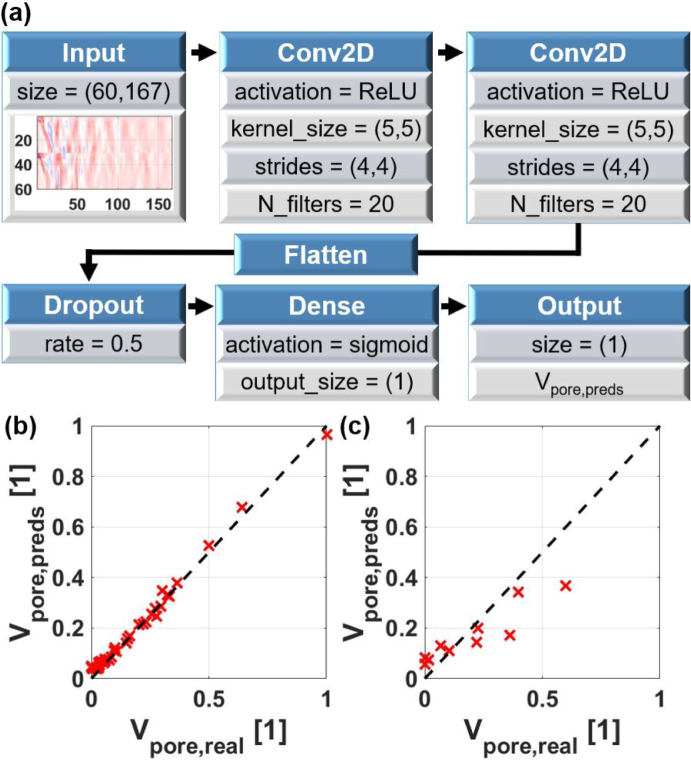
(**a**) Architecture of the neural network (NN) used for predicting the volume of air inclusions from LUS B-scans. (**b**) Correlation with a correlation coefficient of 1.00 between pore volume $V_{pore,real}$ and pore volume predictions $V_{pore,LUS}$ from LUS B-scans by the NN for the 38 samples used for training. (**c**) Correlation with a correlation coefficient of 0.93 between pore volume $V_{pore,real}$ and pore volume predictions $V_{pore,LUS}$ from LUS B-scans by the NN for the 10 test sample.

A framework capable of handling numerous parameters and requiring no initial conditions to be specified are artificial intelligence (AI) methods such as neural networks (NN). Therefore, due to the complex influence of the unique weld seam geometry on the B-scans, this work employs a NN to identify the regions in the B-scans where structures from air inclusions are dominant. The NN under consideration was implemented in Python using Keras (TensorFlow) and had a relatively simple structure, as illustrated in Fig. 8(a), comprising an input layer, followed by two convolutional layers with ReLU activation functions, a flatten layer, a dropout layer, and a dense output layer with a sigmoid activation function. Furthermore, the following regularization terms were utilized for the convolutional and dense layers: kernel (L1: 10^−4^, L2: 10^−4^), activation (L2: 10^−5^), and bias (L2: 10^−4^). The root mean squared error was utilized as the loss function in addition with the Adam optimizer with a learning rate of $4\cdot 10^{-4}$. The NN was trained for 100 epochs with a batch size of 1.

The two B-scans, with the two different measurement configurations ic and oc of one weld seam, stacked on top of each other were used as the input for this NN. Additionally, the B-scans were normalized to values ranging from minus one to one and were down-sampled in the time domain by averaging 150 consecutive data points, resulting in a sampling rate of 60 ns and a Nyquist frequency of 8.33 MHz. As previously discussed, low pass filtering with such a cut-off frequency does not influence the results. Down-sampling in the time domain and stacking of the two detection positions on top of each other results in an input size of (60, 167) for the neural network (see Fig. 8(a)). The output of the NN is a number between zero and one, corresponding to the pore volume prediction ($V_{pore,preds}$). As labels, the normalized total pore volume extracted from the CT data ($V_{pore,real}$) was used, with values ranging from zero to one. In the initial trial, 38 B-scans, corresponding to approximately four-fifths of the data set, were allocated for training with the remaining 10 samples designated for testing. The allocation of individual measurements to either the training or test data set was conducted randomly.

Fig. 8(b) displays the performance of the NN on the training set for an ideal splitting between training and test data. As can be seen a perfect correlation of 1.0 was achieved between the predicted ($V_{pore,preds}$) and the real, normalized pore volume values ($V_{pore,real}$). This high correlation was not unexpected, given that the model utilizes 11141 parameters and non-linear functions, which allow for the fitting of arbitrarily complicated functions to a certain extent. Of greater interest was the performance on the unseen test data. The correlation between the predicted values of the test data ($V_{pore,preds}$) and the real pore volumes ($V_{pore,real}$) is depicted in Fig. 8(c), showing a high correlation of 0.93. These values are significantly higher than those achieved with the standard techniques (see Fig. 7), indicating that the NN can more efficiently extract regions from the B-scans where information about the inner quality of the weld seams is stored. Additionally, as is typical for an imbalanced data set, it can be observed that the model underestimates the large pore volume values.

As the data set of 48 samples is still relatively modest in size, and to ensure that the model does not overfit the data, the NN model is evaluated against a control set where all labels (($V_{pore,real}$)) have been randomly permuted. By employing such statistical analysis as suggested by Flexer et al. [39], it can be ascertained whether the model is genuinely learning to estimate the pore volume in the weld seams by examining the B-scans, or if any arbitrary correlation can be discerned from the B-scans. The learning and testing procedure for both data sets was repeated a thousand times and after each learning and testing procedure the model was deleted, redefined and compiled. Additionally, in each repetition, the data set was randomly split into training and testing sets, and a new mixing of labels was applied in the case of the second (mixed) data set.

**Fig. 9:**
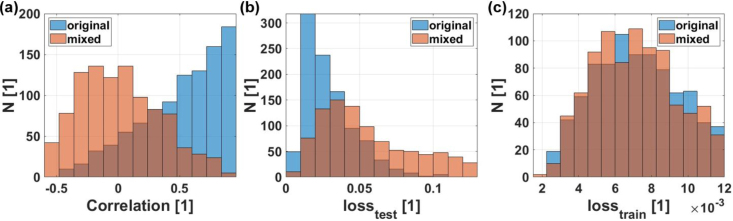
Generalization performance comparison of the NN trained with original labels with one that has been trained on a data set with randomly mixed labels. Overlapping regions of both statistics are indicated by dark brown color. In (**a**) the statistical distributions of the correlation coefficient between predictions and real total air inclusion values are plotted. In (**b**) the statistical distribution of the mean squared error loss on the test set and in (**c**) the statistical distribution of the mean squared error loss on the training set.

A comparison of the statistics for both data sets is illustrated in Fig. 9. In image (a) histograms of the correlation coefficients between real ($V_{pore,real}$) and predicted ($V_{pore,preds}$) pore volumes are plotted for original (blue) and mixed labels (red). With the original labels, the mean (0.53) and median (0.59) correlation is much higher in comparison with the mixed case, where both these values are approximately zero. This means that it is easier for the model to generalize the learned rules to the unseen data when the labels are not randomly mixed. In Fig. 9(b) it can be observed that the loss (root mean square) on the test set is significantly lower in the case with the original labels. On the other hand, Fig. 9(c) shows that there is not much difference between the case with the original labels and with the mixed labels for the loss on the training set, meaning that in both cases the NN is able to learn rules with which the training set can be explained. The difference is that, for the case with the original labels, these rules can be generalized to the unseen data, which is not the case for the mixed labels.

**Fig. 10:**
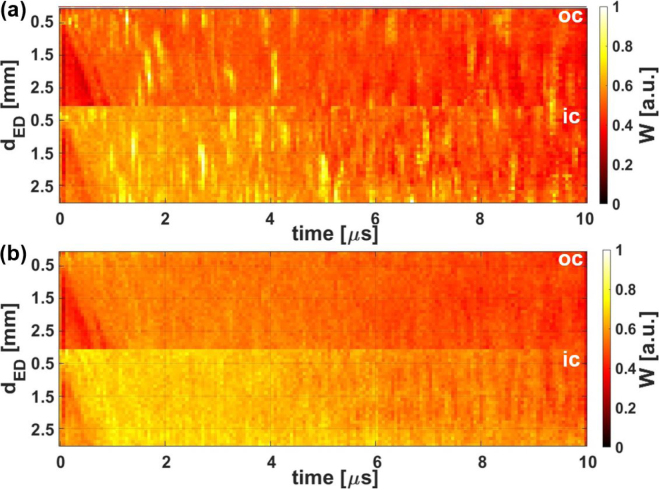
(**a**) Weight (W) of the NN for each pixel of the B-scans when predicting the quality of the weld seams with the original labels averaged over 1000 runs. (**b**) Weight (W) of the NN for each pixel of the B-scans when predicting the quality of the weld seams with the mixed labels averaged over 1000 runs. ic and oc correspond to the two measurement configurations and $d_{\mathrm{ED}}$ to the distance between excitation and detection position.

In Fig. 10(a), the average value over the 1000 training and testing iterations of the weights (W) of the NN for each pixel of the B-scan is plotted, and thereby the regions in the B-scans that are used by the NN to extract information about pores in the interior of the weld seams are highlighted. In Fig. 10(b), the average weights (W) for the case with the mixed labels are plotted, showing a much smoother distribution compared to the case with the original labels. This indicates that the NN can filter out regions containing more information about the total pore volume in the hair pin weld seams.

**Fig. 11:**
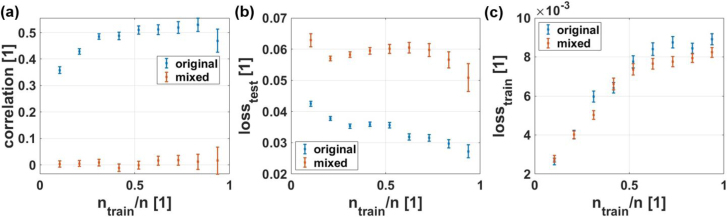
Dependency of NN performance on ratio of samples used for training $n_{\mathrm{train}}$ to number of samples n = 48 averaged over 200 runs. In (**a**) the dependency of the mean correlation coefficient between predictions and true values are plotted for the case with the original (blue) and the mixed labels (red). The error bar indicates the standard error of the mean. In (**b**) the dependency of the mean loss on the test set and in (**c**) the dependency of the mean loss on the training set are displayed.

The mean value of the correlation between predicted and real pore volume of the test data depends on the ratio of training to test data, as shown in Fig. 11(a) for nine different ratios between 0.1 and 0.94. Furthermore, the respective dependencies of the loss on test set (b) and the loss on the training set (c) are depicted. For these statistics, 200 randomly chosen splits into training and test sets were used. Overall, it can be seen that with increasing size of the training data set, the mean loss on the test set decreases, and the mean loss on the training set increases. The mean correlation between the predictions on the test set and the real values is centered around zero for the mixed data set and increasing up to mean values of 0.55 at a ratio of 0.83 for the original data set. It is noteworthy that, starting at a ratio of about two-thirds, the error bars (standard error of the mean) of both the correlation and the loss on the test set increase, indicating a stronger dependency on the splitting between training and test data due to the smaller amount of test data and the underrepresentation of the samples with large pore volumes. Furthermore, it is notable that the error bar is larger in the mixed case than in the original case for both the mean correlation and the mean loss on the test set. This higher variability of the predictions is due to the higher randomness in case of the mixed labels. This cannot be observed for the loss on the training set, which additionally indicates that the generalization in the mixed case is not given.

## 4 Discussion and conclusions

These results demonstrate the potential for estimating the pore volume in hair pin weld seams from LUS B-scans. The ground truth consisted of the pore volume values extracted from CT data. Standard methods based on the analysis of back scattered ultrasound energy were found to be insufficient to estimate the air inclusion volumes from the B-scans due to the complex and unique geometry of each weld seam. This leads to interferences between backscattered ultrasound from inclusions and from structures that arise due to the particular weld geometry. In principle, regions in the B-scans can be identified where information about the presence of pores is contained. However, this results in a multi-parameter optimization problem that lacks generalizability. As an alternative, we propose AI-based methods, which were successfully employed to estimate pore volumes from the LUS B-scans.

The correlation coefficient between the pore volume estimations of the NN from LUS B-scans and the real pore volume extracted from the CT data found in a statistical analysis with median values of up to 0.6 shows already high potential for inline porosity estimation of hairpin welds. Further improvement can be expected by extending the training data set through additional measurements or through numerical simulations. Another approach would be to integrate data from methods described in the introduction, which analyze optical measurements that can cope with the limited sample accessibility or other process parameters of the laser welding process in the NN. By doing so, the NN could also be trained to identify weld seams with geometrical defects, as shown previously in the literature. Furthermore, a more advanced NN can be used to enhance the results of the pore volume estimation. Once trained, the NN can provide a very fast tool for porosity estimation in hairpin welds.

Future investigations will involve a more detailed examination of the influences of the unique weld seam surface and how information obtained with an optical system about the outer topology can be used to improve the defect detection. For instance, the impact of alterations in the angle of incidence of the excitation beam on the sample surface during scanning, along with the resultant changes in absorbed light energy, could thereby be incorporated into the analysis. Furthermore, as regions in the B-scans have been identified which are more significant when trying to estimate the pore volume, this information could be used to reduce the LUS measurement time by reducing the number of excitation and detection position pairs. This analysis of favorable excitation–detection position pairs could be further enhanced through numerical simulations following the scheme introduced in Saurer et al. [29] for aluminum weld seam models, which takes the influence of the geometry (curvature of the surface) on the ultrasound radiation pattern into account. Additionally, B-scans generated by numerical simulations considering the boundary conditions and failure settings can be used to expand the data set for training the NN. All of these findings can be used for efficient, non-contact defect detection and porosity estimation in samples with highly complex geometries where it is hard to define a reference sample, like in hairpin welds.

## CRediT authorship contribution statement

**Markus Saurer:** Writing – original draft, Visualization, Validation, Methodology, Investigation, Data curation, Conceptualization. **Guenther Paltauf:** Writing – review & editing, Supervision, Resources, Conceptualization. **Oliver Spitzer:** Validation, Resources, Investigation. **Tobias Reitmayr:** Writing – review & editing, Validation, Resources, Investigation. **Gordana Djuras:** Validation, Investigation, Data curation. **Birgit Kornberger:** Validation, Investigation, Data curation. **Ulrike Kleb:** Writing – review & editing, Validation, Project administration, Investigation, Funding acquisition. **Robert Nuster:** Writing – review & editing, Supervision, Resources, Project administration, Investigation, Funding acquisition, Conceptualization.

## Declaration of competing interest

The authors declare that they have no known competing financial interests or personal relationships that could have appeared to influence the work reported in this paper.

## Data Availability

Data will be made available on request.

## Appendix A Supplementary data

Video S1 Different views of numerically simulated 3D elastic wave propagation in hairpin welds with and without air inclusions.
